# The Unintended Benefits of the Conservation Reserve Program for Air Quality

**DOI:** 10.1029/2022GH000648

**Published:** 2022-10-11

**Authors:** Douglas A. Becker, Alexander Maas, Jude Bayham, James Crooks

**Affiliations:** ^1^ Agricultural Economics and Rural Sociology University of Idaho Moscow ID USA; ^2^ Colorado State University Fort Collins CO USA; ^3^ Division of Biostatistics and Bioinformatics National Jewish Health Denver CO USA

**Keywords:** fine particulate matter, agricultural conservation

## Abstract

The link between agriculture and air pollution is well‐established, as are the benefits of the US Department of Agriculture's Conservation Reserve Program (CRP). However, little research has linked CRP to air quality directly. This study aims to address this gap by modeling the relationship between CRP and fine particulate matter (PM_2.5_) concentrations at the county level from 2001 to 2016. Several econometric models are estimated with panel data while controlling for drought, population, and wildfire. Results show that CRP has a statistically significant negative effect on PM_2.5_ concentrations. Using estimates from this model, we project an avoided 1,353 deaths, 1,687 deaths, and 3,022 deaths nationally in 2008 relative to three different counterfactual scenarios: all CRP acreage placed under cultivation, increased drought, and a combination of the first two. The value of the avoided mortality is estimated to be $9.5 billion, $11.8 billion, and $21.2 billion, respectively. These findings provide evidence that CRP may generate economic gains in terms of avoided mortality, well above the cost of the program.

## Key Points

- Conservation Reserve Program (CRP) enrollment is associated with lower fine particulate matter concentrations
- The improved air quality from CRP results in fewer related deaths
- CRP pays for itself many times over in saved lives from reduced particulate matter

## 1 Introduction

Agricultural activity is known to contribute to the anthropogenic emission of air pollutants via the application of fertilizers and the use of farm machinery, including greenhouse gasses (Carlson et al., 2017) and hazardous air pollutants such as nitrous oxides and sulfur oxides (Almaraz et al., 2018). Some species of particulate matter, such as ammonium ion and nitrate, are directly emitted by agricultural activities (Paulot & Jacob, 2014), while other pollutants derived from agriculture result from secondary atmospheric reactions. Ammonia (Cooter et al., 2012) and volatile organic compounds (VOCs) (Pozzer et al., 2017) for example, are emitted via fertilizer application, and react atmospherically to create the secondary pollutants, particulate matter and ozone (Chandler et al., 2004). In the case of particulate matter, agriculture constitutes anywhere from 25% of total anthropogenic particulate matter in India to 55% in Europe (Bauer et al., 2016). Similar estimates for the United States are not currently available, but the results presented herein suggest conventional agriculture may be a strong contributor to PM in the US as well.

Air pollution from agricultural activity (and non‐agricultural sources) is harmful to human health. Both coarse and fine particulate matter are known to cause adverse health outcomes (including asthma, cardiovascular disease, and cancer) and increase the probability of mortality (Burnett et al., 2018; Dai et al., 2014). Particulate matter specifically attributable to agriculture is associated with an observed increase in multiple types of morbidity (Loftus et al., 2015) and mortality (EPA, 2019; Giannakis et al., 2019; Paulot & Jacob, 2014; Pozzer et al., 2017).

Drought can influence agriculture and agricultural air pollutant emissions significantly. Ground that is drier is more likely to generate dust, leading to worsened air quality (Achakulwisut et al., 2018). Rain captures dust particles, and the lack of rain causes the release of more dust into the atmosphere (Lau, 2014). The nitrogen applied in fertilizers becomes more volatile and reactive in an environment with elevated atmospheric temperatures and less cloud cover (Christopher & Gupta, 2010). The health impacts of PM may therefore be amplified by drought, resulting in disproportionately higher morbidity and mortality (D'amato et al., 2016). Thus, a better understanding of the relationship between PM and agriculture will be crucial in designing policies under future climate conditions.

Because of the well‐documented relationship between PM and health, local, state, and federal agencies have implemented numerous laws, regulations, and policies to reduce air pollutant emissions. The most notable of these regulations is the Clean Air Act, originally passed in 1963 and amended most recently in 1990 (Popp, 2003). Air quality has improved markedly during that time, with reductions in some air pollutant concentrations exceeding 90% (LaCount et al., 2021). In addition to regulations such as the Clean Air Act, land‐use policy has significant implications for emissions and human health. Although not an explicit objective, agricultural policy in the form of conservation and environmental quality programs may also serve as a policy avenue for air pollution reduction.

The US Department of Agriculture (USDA) operates national agricultural conservation programs, the most prominent of which is the Conservation Reserve Program (CRP), designed to aid farmers in efforts to preserve, conserve, and improve the natural environment (Hellerstein, 2017). Such programs provide benefits such as reduced erosion, improved water quality, and restored wildlife habitat (Johnson et al., 2016; Ribaudo et al., 1989). Though it is not a stated objective of the program, the CRP may also improve air quality by decreasing the use of heavy machinery and the application of synthetic fertilizers. While these vectors of air pollutants are intuitive, no empirical work has estimated the observable effect of CRP enrollment and drought on air quality at the national and regional scale.

The proceeding analysis therefore attempts to quantify (a) the effect of CRP enrollment on PM_2.5_ concentrations, (b) the effect of drought on PM_2.5_ concentrations, and (c) the benefits, measured through avoided mortality, of increases in CRP enrollment and lessened drought conditions. No prior studies have empirically estimated the effect that CRP has on PM_2.5_. Considering the scale and cost of the CRP program—as many as 5.3 million acres and $1.9 billion dollars in 2020 (USDA, 2021)—a better accounting of its total costs and benefits is crucial. This study fills these gaps.

## 2 Methods

### 2.1 Estimating the Effect of CRP and Drought on PM_2.5_

This study uses county‐month as the unit of observation in an ecological panel study design. The study period covers the years 2001–2016, the years for which a complete data record exists for all relevant dependent and explanatory variables. This timeframe includes both a period of increasing CRP enrollment (2001–2008) and decreasing enrollment (2009–20016) to ensure that inferences are not simply a result of contemporaneous time trends. The geographic extent of the analysis includes the contiguous US, though counties with missing data (677) or zero enrollment in CRP (153) throughout the 17‐year timeframe are removed from the sample such that statistical models are estimated using the remaining 2,287 counties.

## 3 Data

### 3.1 Dependent Variable

The dependent variable of interest is the average monthly fine particulate matter (PM_2.5_) mass concentration in micrograms per cubic meter of air for each county. All species of PM_2.5_ are included in this data set. While PM_10_ and specific PM_2.5_ species also have implications for human health (Adar et al., 2014; Yang et al., 2019), consistent measurement at the county level is not available for a consistent timeframe in which sufficient variation in CRP enrollment exists. As such, our analysis is limited to PM_2.5_ mass. Since there is a dearth of air quality monitoring stations present throughout the rural United States (only 773 of the 3,142 counties in the United States have stations), daily county‐level modeled PM_2.5_ concentration data were collected from the Centers for Disease Control's (CDC) National Environmental Public Health Tracking Network (CDC, 2021a, 2021b) and were aggregated by year‐month. The CDC developed this data set with the Environmental Protection Agency's down‐scaled air quality model (Wang et al., 2018), which, by regridding Moderate Resolution Imaging Spectroradiometer satellite data with the EPA's Community Multi‐Scale Air Quality model and incorporating atmospheric and meteorological conditions from the North American Regional Reanalysis program, corrects for modeling bias via calibration by monitored air quality data.

### 3.2 Explanatory Variables

The independent variable of interest reflects CRP enrollment and drought conditions. CRP enrollment is included as the percent of total arable land enrolled in the CRP. We calculate total arable land as the maximum of the sum of all acres planted and all acres enrolled in CRP for each county‐year. This normalization was chosen to account for the substantial size differences in agricultural acreage across counties. The denominator is constant for a county across all years, but the numerator varies based on newly enrolled or expired CRP contracts. This fraction is multiplied by one hundred such that the CRP variable can range from 0 to 100. The CRP acreage data used in this study come from the Farm Service Agency's Conservation Reserve Program Statistics portal (FSA, 2021).

To capture drought, which can significantly influence air quality (Achakulwisut et al., 2019), the Palmer Drought Sensitivity Index (PDSI) was included in the model. PDSI was chosen ahead of other potential drought metrics because it was designed to account for the impact of land memory on drought conditions (NCAR Climate Data Guide). Additionally, PDSI accounts for locational differences, unlike Standardized Precipitation Indexes that may have similar scores in two locations but does not capture true water deficit (due to their innate differences) The PDSI is computed from precipitation volume, air temperature, and soil moisture content and has been evaluated as an accruable and reliable measurement of drought and drought risk (Osborn et al., 2017). The index used was sourced from the Climate Engine, which uses daily county‐level meteorological data from gridMET (Abatzoglou, 2013), and ranges between negative eight and eight, though more extreme values are possible.

### 3.3 Control Variables

#### 3.3.1 Temporal Variables

The presence of seasonality and overall trends in both PM_2.5_ concentrations and agricultural activity require additional control variables. A vector of monthly dummy variables is included in the model to account for seasonal changes in PM_2.5_ and agricultural production. The long‐run effects of air pollution policies (e.g., revisions to the U.S. EPA's National Ambient Air Quality Standard for PM_2.5_), climatic conditions (e.g., El Nino), and macroeconomic conditions are controlled via annual dummy variables for each year of the sample.

#### 3.3.2 County‐Specific Variables

We use county‐specific fixed effects (i.e., county‐specific intercepts) to control for all time‐invariant factors such as geographic factors, industry‐type, elevation as well as unmeasurable features of culture that influence agricultural practices but do not vary over the study period. The use of fixed effects is a parsimonious approach to mitigating potential omitted variables bias. In addition to these fixed effects, time‐varying continuous variables are included to control for variation in drought severity, urbanization, and wildfire. While this list of control variables is not exhaustive in describing the formation and dispersion of fine particulate matter, the inclusion of both geographic and temporal fixed effects provides some assurance that our estimates are accurately capturing the effect of CRP and drought.

#### 3.3.3 Population

The level of economic and industrial development in any county is inversely related to the amount of land devoted to agriculture (Satterthwaite et al., 2010) and influences air quality (McCarty & Kaza, 2015). As such, we control for the level of urbanicity in a county. The National Center for Health Statistics (NCHS) urban‐rural classification scheme classifies from most to least urban based on the population of the county; for example, counties with a population of one million or more are classified as most urbanized. However, the NCHS scheme is only available for certain years (2006, and 2013), and more temporally complete urbanization data were important to capture annual population fluctuations. Therefore, we chose county population as an alternative representation of county urbanization, as population often accurately captures the level of development in a county (Ingram & Franco, 2012). While we use population in our preferred specification, we also estimated the model using the NCHS urban‐rural classification and found our results are not significantly affected. The county population data used in this study come from the US Census American Community Survey annual population estimates (US Census Bureau, 2021).

#### 3.3.4 Wildfires

Wildfires are acute events responsible for substantial particulate matter emissions that may not be captured by the fixed effects controls described above. Wildfires were included in the model as acres burned by county‐month as an approximation for wildfire smoke presence and intensity. The total acres burned in wildfires was calculated for each county by summing the acreage of all reported wildfires in each month. This does result in some double counting of fire acreage since a fire can burn across multiple months. Additionally, the size of a fire in a county may not correlate perfectly with PM emissions, nor would it account for inter‐county transport of smoke, but this operationalization balances the importance of controlling for wildfires and the limitations of data availability and computational resources. Wildfire acreage data were taken from the US Forest Service Fire Occurrence Database (Short, 2021).

### 3.4 Analysis and Model Choice

The first step in our analytical process was the calculation of descriptive statistics and bivariate correlations to explore simple statistical associations between dependent, independent, and control variables. We then ran multivariate statistical models, starting first with ordinary least squares (OLS). Diagnostic tests were conducted on the OLS model to ascertain if more advanced modeling techniques were required, and the designated models were then executed, including fixed effects (FE) and spatial fixed effects (SFE) models. The analytical approach is described below.

While the OLS model may provide insight, it is a naïve approach that is unlikely to capture the true nature of the data‐generating processes necessary for accurate hypothesis testing. The FE model controls for the many potentially unobservable factors influencing PM_2.5_ and overcomes many of the problematic assumptions embedded in the OLS specification. Using the FE specification allows us to estimate the relationship between predictor and outcome variables within the county and allows for correlation between a county's error term and omitted variables, and controls for time‐invariant factors such as soil characteristics. A random effects (RE) model was also considered. The RE model assumes that individual county effects are uncorrelated with the independent variables, a potentially dubious assumption in this case. Parameter estimates from the FE model are still consistent if individual county effects are correlated with the independent variables. A Hausman (1978) test was used to determine the appropriate model and suggested that FE was preferred to RE. In all specifications, we cluster standard errors at the county level to account for autocorrelation and heteroskedasticity.

### 3.5 Sensitivity Analysis

Fine particulate matter concentrations fluctuate over the course of a year as atmospheric conditions and anthropogenic activities vary. It is therefore important to ensure that predictions of fine particulate matter concentrations are robust to different formulations of the temporal dimensions of a model. As a sensitivity analysis, we ran a generalized additive model (GAM) with cubic spline smoothers on the month and year variables to determine if and how our results differ when time is modeled differently. GAMs with cubic spline smoothers are well‐suited for detecting and controlling the variability in temporal variables (Youngman & Economou, 2017).

### 3.6 Quantifying the Air Quality Impacts of CRP and Drought

Non‐accidental mortality attributable to specified changes in CRP and PDSI are estimated using the formulas and parameters developed by Burnett et al. (2018). The concentration‐response function published in that work is formulated using results from 41 cohorts across 16 countries and has been used previously in U.S.‐specific health effects research (e.g., Mailloux et al., 2022). The population‐wide hazard ratio, predicted annual average fine particulate matter concentrations, and baseline non‐accidental county mortality incidence counts were used to estimate excess mortality in three counterfactual scenarios; Scenario 1 (all CRP removed, i.e., all available farmland is under cultivation); Scenario 2 (a decrease of 4 on the PDSI scale); and Scenario 3 (a combination of Scenarios 1 and 2). Results from these scenarios are compared to the estimated mortality of actual CRP and PDSI measures for 2008, the year of maximum CRP enrollment (and before much of the fallout from the Great Recession) during the study period. Baseline mortality counts were taken from the CDC's Wide‐ranging Online Data for Epidemiologic Research (WONDER) database (CDC, 2021a; 2021b). As such, the avoided mortality for each scenario is equivalent to excess mortality based on predicted PM changes of each counterfactual scenario compared to the baseline.

Excess mortality estimates are then monetized using the Value of a Statistical Life (VSL) of $7 million. Although we acknowledge the ongoing ethical and empirical debates around the VSL (Alberini et al., 2004; Cai et al., 2021; Van Wee & Rietveld, 2013), we elect an unconditional $7 million dollar estimate based on a recent meta‐analysis of meta‐analyses by Banzhaf (2021).

## 4 Results

### 4.1 Descriptive Statistics, Distributions, and Bivariate Correlations

**Figure 1:**
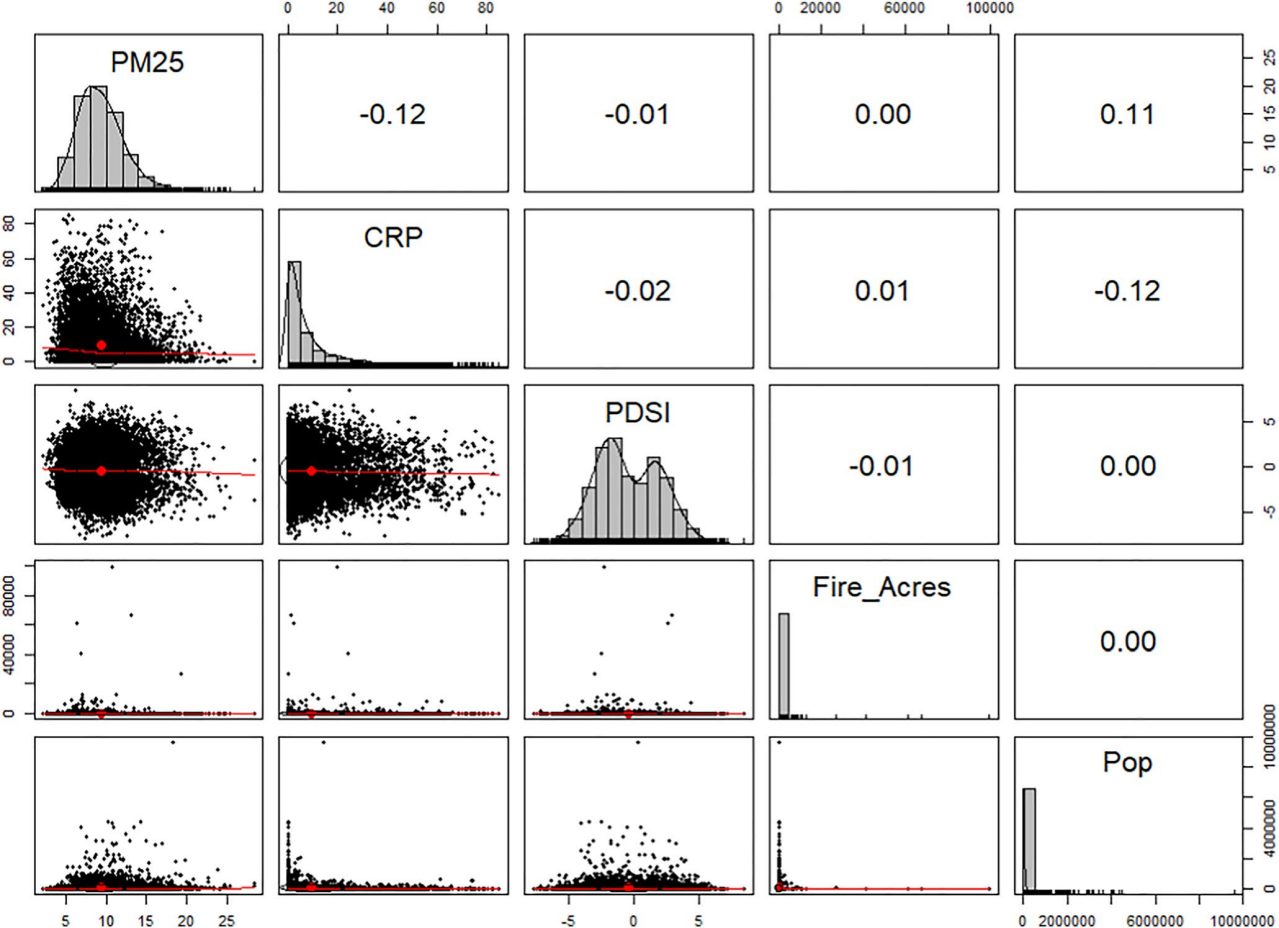
Graphical matrix displaying histograms with distribution curves (diagonal), scatterplots with loess curves (lower left), and bivariate correlations (upper right) graphs on a sample of 10,000 observations drawn from the study data.

Across the entire sample PM_2.5_ concentrations by county‐month have a mean of 9.5 μg/m^3^, and a standard deviation of 2.1 μg/m^3^. The percentage of arable land that was enrolled in the CRP has a mean of 10.1%, and a standard deviation of 12.5%. Full descriptive statistics for all variables can be seen in Table 1. The unconditional bivariate Pearson correlation coefficient between PM_2.5_ and CRP was −0.12 (*p* < 0.01), indicating a small statistically significant relationship between the variables. The scatterplot between CRP and PM_2.5_ (Figure 1, second from top left vertically) also suggests a slight downward trend in county PM_2.5_ concentrations as CRP acreage increases.

**Table 1:** Descriptive Statistics for the Dependent, Independent, and Control Variables

Variables	Mean	Std. Dev.
PM_2.5_ (micrograms per cubic meter)	9.5	2.1
CRP (%)	10.1	12.5
PDSI (−8 to 8 scale)	−0.4	2.5
Fire Acres	0.1	3.1
Population (1,000s)	70.2	249

*Note*. *N* = 439,104 observations; 2,287 unique counties.

### 4.2 Model Results

In the fixed‐effects model the CRP share of arable land exhibits a statistically significant negative effect on particulate matter with a point estimate of −0.020 (Table 2). The fixed‐effects model yields an adjusted *R* ^2^ of 0.35 (within variation). Qualitative results are consistent across the suite of models used in our analysis but point estimates of CRP on PM_2.5_ vary; −0.037 (*p* < 0.05) in the OLS model and −0.020 (*p* < 0.05) in the FE model. Full fixed‐effect model results can be seen in Table 2. The spatial fixed‐effects model did not properly fit the data because the model was unable to converge and is therefore not presented as part of the main results of this study. The results of the Spatial‐autocorrelation‐Fixed Effects (SAC‐FE) model can be seen in Supporting Information S1.

**Table 2:** Full Sample Model Results for Three Model Specifications, for 439,104 Observations From 2,287 Counties

Variables	OLS	FE	GAM CS
Coefficients			
CRP	−0.037***	−0.020***	−0.037***
PDSI (−8 to 8 scale)	−0.022***	−0.046***	−0.014***
Fire Acres	0.002	0.022***	0.000
Population (1,000s)	0.001***	−0.005***	0.000
Model parameters and fit statistics			
Adj./Pseudo *R* ^2^	0.25	0.35	0.25

*Note*. The dependent variable is PM_2.5_ in micrograms per cubic meter. The fixed effects (FE) model includes county, year, and month fixed effects. The generalized additive model includes cubic spline smoothers on the year and month variables, and both smoothing terms were statistically significant at the 0.001 level. ***p < 0.001, **p < 0.01, *p < 0.05.

As expected, drought significantly increases particulate matter concentrations according to our fixed‐effects model, though coefficients on PDSI are not directly comparable to CRP since the underlying variable range is much smaller (−8 to 8 vs. 0 to 100). Results suggest that annual average PM_2.5_ concentrations increase by as much 0.5 μg/m^3^ from very wet to very dry years. Fire also has a strong positive relationship with PM_2.5_. Population (our measure of urbanization) by comparison has a negative relationship with PM_2.5._ Note that the population coefficient is positive in the OLS specification indicating that areas with higher population do have higher PM2.5 concentrations. However, the county fixed effects explain most of that variation, and within a county, places with growing populations have lower PM2.5 concentrations which may reflect more stringent regulations in growing counties.

### 4.3 Sensitivity Analysis

The GAM with cubic spline smoothers did not yield results that were substantively different from the fixed‐effects model. The beta coefficient of the CRP variable was found to be −0.037 (*p* < 0.05) in the GAM, identical to the OLS model and similar in direction and magnitude to the fixed‐effects model. The other coefficients followed this pattern of mirroring the main results models as well. Full results for the GAM with cubic spline smoothers can be seen in Table 2 along with the other model results.

### 4.4 Quantifying the Impacts of CRP and Drought

**Figure 2:**
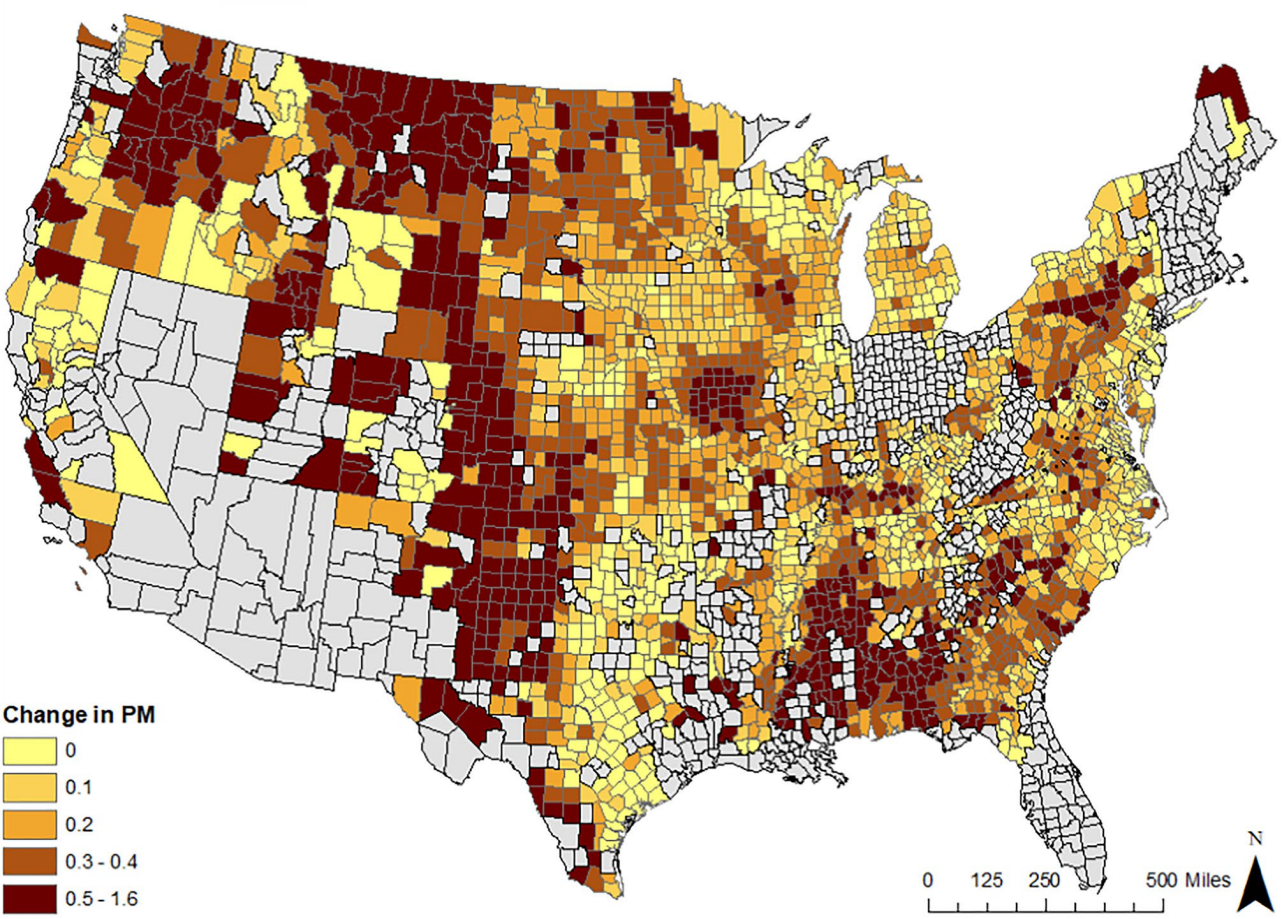
Reduction in county‐level PM_2.5_ μg/m^3^ calculated as the difference between the zero‐Conservation Reserve Program (CRP) scenario (counterfactual) and the high‐CRP‐enrollment year of 2008 (observed) as per the predicted PM from the fixed‐effects model.

Mean mortality attributed to specific changes in CRP and PDSI is estimated, based on predicted PM concentrations from the FE model, at 1,353 deaths in Scenario 1 (CRP = 0), or 0.05% of all deaths in the United States in 2008. Extreme drought (Scenario 2) accounts for 1,687 deaths. As many as 3,022 deaths are estimated in Scenario 3 (CRP = 0 and PDSI ‐4). The full results, including lower and upper death estimates (95% Confidence Intervals), are found in Table 3. Assuming a VSL of $7 million, these estimates suggest a loss of $9.5 billion in Scenario 1, $11.8 billion in Scenario 2, and $21.1 billion in Scenario 3. Figure 2 displays the spatial distribution of the estimated change PM_2.5_ in Scenario 1 in 2008 compared to the baseline prediction, and full mortality and economic valuation estimates, including 95% confidence intervals, are found in Table 3.

**Table 3:** Lower, Mean, and Upper Bound Estimated Mortality and Economic Valuation of Mortality in Three Scenarios; No CRP, a PDSI Reduced by Four, and a Combined No‐CRP Four‐PDSI Scenario Nationwide in 2008, Compared to the Baseline Scenario (Unmodified CRP and PDSI)

Mortality			
Scenario	Lower 95% CI	Mean	Upper 95% CI
1 (CRP = 0)	1,040	1,353	1,647
2 (PDSI −4)	1,299	1,687	2,052
3 (CRP = 0 and PDSI −4)	2,326	3,022	3,676
Economic valuation (billion USD)			

Scenario	Lower 95% CI	Mean	Upper 95% CI
1 (CRP = 0)	7.3	9.5	11.5
2 (PDSI −4)	9.1	11.8	14.4
3 (CRP = 0 and PDSI −4)	16.3	21.2	25.7

## 5 Discussion

### 5.1 Interpretation of Findings

Our models show a statistically significant and negative association between the percent of arable land that is enrolled in CRP and concentrations of fine particulate matter in a county. Although the magnitude of the association is small, for example, −0.020 μg/m^3^ PM_2.5_ per 1% of arable land enrolled in CRP under the FE model, the negative trend confirms the expectation that CRP reduces particulate matter concentrations. This reduction in PM_2.5_ translates to a decrease of 1,353 deaths per year attributed to CRP enrollment.

These findings affirm the results of previous studies that have reported a link between agricultural production and air pollution (Bauer et al., 16; Chandler et al., 2004; Paulot & Jacob, 2014) and highlight benefits of the Conservation Reserve Program to air quality (Johnson et al., 2016). Our results contribute to these existing studies by using a statistical model with actual CRP enrollment and modeled PM_2.5_ data to elucidate the relationship between the two factors while controlling for key environmental and demographic confounders.

Although our results suggest that CRP does exert some influence on air quality, it is possible that this is achieved through the removal of fertilizer‐intensive cropland from production rather than the conservation and other components of the CRP. Some of the land enrolled in CRP is replanted elsewhere (Wu, 2000), sometimes as crops that require larger amounts of fertilizer. Furthermore, CRP acreage has been decreasing in recent years as cropland planted with fertilizer intensive crops such as corn is increasing (USDA, 2021).

Additionally, we found a large association for the PDSI variable (*β* = −0.046, *p* < 0.05) in the fixed‐effects model compared to that of CRP (*β* = −0.020, *p* < 0.05). And, as Table 3 show, the mortality and economic value attributable to a constant change in PDSI are larger than when all CRP is removed from PM concentration predictions. A reverse‐causality, wherein atmospheric PM affects drought and drought affects PM, exists between the two factors (Achakulwisut et al., 2019), indicating a complicated physical‐chemical interactivity. Furthermore, Berman et al. (2017) found positive associations between mortality and drought. Although the examination of the effect of drought on air quality is not the main objective of this study, it is an important finding that suggests that drought is a key driver of agricultural air pollution, and that drought may influence mortality via its effect on PM_2.5_.

### 5.2 Strengths and Limitations

The first strength of this study is the direct analysis of CRP enrollment acreage and PM_2.5_ concentrations, to our knowledge the first study to do so. A second major strength is the employment of fixed‐effects models to adjust for factors that were unfeasible or impossible to account for, although we were able to control for important variables such as drought risk, wildfires, urbanization, and active farming seasonality. Third, our study period consists of 16 years of data across more than 22 hundred counties. This enables us to draw conclusions about the relationship between CRP and PM_2.5_ without the outsize influence of any single county or year, so outliers cannot be said to be responsible for the nature of the relationship.

There are also limitations to this study. Our analysis is not causal. Our study design is not able to identify the mechanisms through which CRP reduces air pollution. This study does not aim to make causal claims, though; given that this is the first study that we know of that relates CRP acreage to fine particulate matter concentrations, our epistemological objective is to discern if any empirical relationship at all exists between the two factors.

A second important limitation is the size of the unit of analysis. Counties can be many hundreds or even thousands of square miles in area with a myriad of landscapes, land uses, and crop types along with disparate and varying environmental conditions. As a result, our analysis might not capture the variability in features, namely land cover and agriculture practices, that could be more precisely measured with a smaller unit. The availability of data for both the dependent and independent, as well as control, variables constrained our study design.

A third limitation is that this study did not directly model or estimate the transport of agricultural emissions from one county to another given observed wind direction and weather patterns. We did attempt to control for spatial spillovers in PM_2.5_ between counties based on spatial adjacency under the SAC‐FE model (Supporting Information S1), but that model did not converge and cannot be considered to fit the data.

Fourth, while acres burned is included in the models to account for fire emission's impact on ambient PM_2.5_ concentrations, it is worth noting that the toxicity of fire derived PM_2.5_ is thought to increase through oxidation the longer the particles are airborne (Finlay et al., 2012; Wegesser et al., 2009). As such, wildfires may have enhanced mortality and health impacts, which are neglected in the present analysis.

Fifth, the 95% confidence intervals in the mortality and economic valuation estimates were calculated based solely on the standard deviations of the health effect estimates in Burnett et al. (2018). There is much uncertainty that is not captured in those confidence intervals, such as the uncertainty in the population data and the mortality rates. Furthermore, the concentration‐response function in Burnett et al. (2018) is not the only possible one, and our work does not account for uncertainty in the functional form itself. We also only estimated the change in PM_2.5_ concentrations, mortality, and economic value for a single PDSI scenario, yet there may be significantly different effects on the outcomes associated with different PDSI values, and simply subtracting four from all counties' PDSI values may be facile.

Finally, this study did not estimate relationships between CRP and other ambient air pollutants besides PM_2.5_ mass, notably NO*x*, VOCs, PM10, and PM_2.5_ species. PM_2.5_ has the best‐characterized health impacts among all pollutants as well as the densest monitoring network, making it the natural choice for an initial study.

### 5.3 Policy Implications

This study provides empirical evidence that the CRP improves air quality, building on a list of benefits of the program that includes reduced soil erosion, enhanced water quality, and restored wildlife habitat (Johnson et al., 2016; Ribaudo et al., 1989). We also show that the CRP pays for itself many times over in just the economic value of the avoided lives lost from agricultural air pollution; CRP costs the federal government $2 billion annually (USDA, 2021), while the value of the avoided mortality from CRP is estimated at as much as $21 billion. Our results should be of interest to the US Department of Agriculture, especially in the consideration of whether to expand or contract the Conservation Reserve Program or what new areas should be targeted for CRP enrollment.

### 5.4 Future Research Directions

Because our results are derived from statistical models and county‐level data, further research is called for to more fully understand how conservation programs such as CRP can improve air quality. Of special importance is the need to understand the physical mechanism or mechanisms through which agricultural conservation practices affect the emission of air pollutants and air pollutant precursors. Atmospheric modeling, field studies, and experiments should be conducted to allow for conclusions about a causal relationship if one does indeed exist. Studies with a smaller unit of analysis, which may well require a smaller study area and alternative data sources, would also contribute to our knowledge of the issue. Additional counterfactual scenarios, beyond the three conducted in this study, could further elucidate the impacts of nonlinearities in the associations and in extreme scenarios. Finally, considering the empirical relationship we observed between CRP and PM_2.5_ mass and the substantial derived health benefits, our result suggests a need for further research to estimate exposure changes and benefits attributable to CRP across the full suite of agriculture‐related air pollutants.

## 6 Conclusion

This study aims to fill a gap in the literature on agriculture and air quality by linking Conservation Reserve Program land to fine particulate matter concentrations in a spatial fixed‐effects model. The model results show a statistically significant and negative association between CRP acreage and PM_2.5_ concentrations, thousands of avoided cases of premature mortality, and billions of dollars in value of the lives of those who would have otherwise died if CRP did not exist. Agricultural and conservation policymakers with an interest in expanding the list of the benefits conferred by conservation programs can take note of our results in doing so. Future research, most notably field experiments and atmospheric modeling studies, are needed to confirm our results and delineate the mechanisms by which conservation programs influence air quality.

## Conflict of Interest

The authors declare no conflicts of interest relevant to this study.

## Supporting information

Supporting Information S1

## Data Availability

The data for this project are free and publicly available on the Open Science Framework platform as a comma‐separated values (CSV) file. No registration or permissions are required to access this data file. The DOI link to access the data file is: https://doi.org/10.17605/OSF.IO/R8BAK.
