# Supplementary material for: The Unintended Benefits of the Conservation Reserve Program for Air Quality
Source: Geohealth. 2022 Oct 11;6(10):e2022GH000648. doi: 10.1029/2022GH000648 (PMC9553094; doi:10.1029/2022GH000648)
Supplement: Supplementary file 1 — Supporting Information S1 [file GH2-6-e2022GH000648-s001.docx]

**The Unintended Benefits of the Conservation Reserve Program for Air Quality**

Douglas A. Becker, Alexander Maas, Jude Bayham, James Crooks

**Supplemental Material 1: SAC-FE Model Methods and Results**

Contents

[Methods 1](#_Toc110424453)

[Results 2](#_Toc110424454)

## Methods

The FE model does not account for potential spatial correlation between nearby counties, which may also be present given the nature of the analysis. Using the residuals of the basic FE model, we calculated the Moran’s I statistic (Moran, 1950) to assess the presence and intensity of spatial autocorrelation (p=0.000). Due to the possible presence of spatial correlation, a third model that accounts for this relationship is also considered.

There are numerous ways in which spatial correlation can exist and be modeled; these include standard, spatial autoregressive (SAR) models, spatial Durbin models (SDMs), spatial error models (SEMs), and spatial autocorrelation-error models (SAC) (Belotti et al., 2017). The key difference across these models is the manner in which autocorrelation enters, through the dependent variable, independent variables, or the residual error term. Using methods suggested by LeSage and Pace (2009) and Elhorst (2010b), and operationalized by Belotti et al., (2017), we conduct a series of statistical tests to determine which model specification is most appropriate. First, a SDM was estimated assuming RE and FE, and a Hausman test again rejected the use of RE (p=0.000).

We then tested the SDM specification compared to SAR and SEM specifications. Based on these tests, SDM was the preferred choice. Because the SAC and SDM are non-nested models, we use information criteria to choose between them. We find that the SAC model has lower AIC and BIC scores (AIC=950,152, BIC=950,513 versus AIC=998,501, BIC=999,183). Thus, SAC appears to be the model best suited to capture the spatial correlation of our data.

Results from all three models (OLS, FE and SAC-FE) are included in the results section. However, because fixed effects cannot be consistently estimated simultaneously with spatial interactions, the main results as well as the mortality and value predictions are derived from the FE model. After estimating the effect of CRP and PDSI on PM_2.5_ concentrations, three counter-factual scenarios are created to estimate avoided PM_2.5_-related mortality benefits of CRP enrollment and moderated drought.

## Results

The SAC-FE model produced a coefficient of -0.003 (*p*=0.000) for the CRP variable and -0.031 (*p*=0.001) for PDSI. Note that the coefficient estimate for SAC-FE does not include the indirect effect of neighboring counties. When this effect is included in the estimate, CRP has a total effect of -0.0041 µg/m^3^ per percent enrolled (not shown in table).

**Table S1.** Full sample model results in the SAC-FE model.

| **Variables** | **SAC-FE** |
| --- | --- |
|  |  |
| PDSI | -0.031*** |
|  | (0.001) |
| CRP | -0.003*** |
|  | (0.000) |
| Population | -0.002*** |
|  | (0.000) |
| Fire Acres | 0.005*** |
|  | (0.000) |
| Constant |  |
|  |  |
|  |  |
|  |  |
| Adj./Pseudo R^2^ | 0.17 |
| Observations |  |
| Unique Counties |  |

FE and SAC-FE models include county, year, and month fixed effects.

*** p<0.001, ** p<0.01, * p<0.05
